# Optimization of Molasses and Soybean Meal Content to Enhance Tetramethylpyrazine Yield by *Bacillus* sp. TTMP20

**DOI:** 10.3390/molecules28186515

**Published:** 2023-09-08

**Authors:** Yujia Li, Shanling Gan, Lirong Luo, Wenjing Yang, Lei Mo, Changhua Shang

**Affiliations:** Key Laboratory of Ecology of Rare and Endangered Species and Environmental Protection (Guangxi Normal University), Ministry of Education, Guangxi Key Laboratory of Landscape Resources Conservation and Sustainable Utilization in Lijiang River Basin (Guangxi Normal University), Guilin 541006, China; liyujia@stu.gxnu.edu.cn (Y.L.); ganshanling1999@163.com (S.G.); gxnu202011002019@stu.gxnu.edu.cn (L.L.); yangwenjing0726@163.com (W.Y.); 18894751263@163.com (L.M.)

**Keywords:** *Bacillus* sp. TTMP20, tetramethylpyrazine, molasses, soybean meal, pretreatment, central composite design

## Abstract

Microbial fermentation for the production of tetramethylpyrazine (TTMP) is considered to be the most promising method, and the development of a cheap fermentation substrate is of great importance for large-scale TTMP production. In this study, inexpensive by-products from the food industry, i.e., molasses and soybean meal (instead of glucose and tryptone), were used as substrates for TTMP fermentation. The pretreatment of soybean meal was explored in order to achieve a better fermentation effect. The contents of each component in the fermentation medium were optimized by central composite design (CCD). The optimum contents were as follows: 72.5 g/L of molasses, 37.4 g/L of diammonium hydrogen phosphate (DAP), 53.4 g/L of soybean meal, and 5 g/L of yeast powder. The software predicted a maximum TTMP yield of 1469.03 mg/L, and the actual TTMP yield was 1328.95 mg/L for the validation experiment in the optimum medium. Under the optimum conditions (72.5 g/L of molasses, 37.4 g/L of DAP, 53.4 g/L of soybean meal, and 5 g/L of yeast powder), the actual maximum TTMP yield (1328.95 mg/L) in this study was much higher than the TTMP yield (895.13 mg/L) under the conditions (150 g/L of molasses, 30 g/L of DAP, 30 g/L of tryptone, and 10 g/L of yeast powder) of our previous study published in *Molecules*. In this study, the TTMP yield improved by 48.46%, with decreased molasses (more than half), decreased yeast powder (half) and by-product soybean meal instead of tryptone compared to our previous study. In summary, the cheaper fermentation medium had a higher TTMP yield in this study, which improves the application potential of *Bacillus* sp. TTMP20.

## 1. Introduction

Chuanxiong (*Ligusticum wallichii* Franch) is a traditional Chinese medicine. Its rhizome has the effect of promoting blood circulation and removing blood stasis [1]. So far, more than 200 chemical constituents have been isolated from Chuanxiong, which includes phthalides, terpenes, alkaloids, polysaccharides, and organic acids.

TTMP is an alkaloid from Chuanxiong [2]. Current research has shown that TTMP is effective for the treatment of cardiovascular diseases, including hypertension, coronary heart disease, viral myocarditis, heart failure, and arrhythmias [3]. The efficacy of TTMP in treating other diseases (such as cancer, diabetes, liver damage, kidney damage, and ischemic stroke) has also received more attention [4]. TTMP has also been detected in fermentation products, such as Chinese Baijiu, vinegar, and natto, which are important flavor components.

At present, there are three ways to obtain TTMP: plant extraction, chemical synthesis, and microbial fermentation [2]. Plant extraction mainly uses Chuanxiong, which has shortcomings such as low yield, a cumbersome and time-consuming process, and limited raw material sources, and it cannot meet the huge market demand. So far, the industrial production of TTMP mainly relies on chemical synthesis. Although the yield of chemical synthesis is higher than that of plant extraction, it also has disadvantages, such as severe pollution, high production cost, and an uncontrollable reaction process.

Kosuge et al. successfully isolated TTMP from natto, a traditional Japanese fermented food, and discovered that microorganisms had the ability to synthesize TTMP for the first time [5]. Since then, studies on TTMP production by microorganisms showed rapid development. According to the literature reports, many bacteria and fungi can produce TTMP. Microorganisms producing TTMP include thermophilic actinomycetes, *Bacillus* sp., and *Saccharomyces cerevisiae* [6,7,8]. *Bacillus*, due to its wide distribution and strong resistance to various environments, is a kind of microorganism that has been extensively studied. Among TTMP-producing strains, *Bacillus* is the dominant kind. Many studies have focused on TTMP-producing *Bacillus* strains, including *Bacillus* mutants [9], *Bacillus subtilis* [10], *Bacillus amyloliquefaciens* [11], and *Bacillus coagulans* [12].

The ability of microorganisms to produce TTMP is limited, so current studies on the microbial production of TTMP mainly focus on increasing TTMP production. Multiple methods, such as genetic engineering, metabolic engineering, and mutation, have been used to increase TTMP yield [13]. Various fermentation strategies have been applied to the study of the microbial production of TTMP. Larroche et al. and Besson et al. added a precursor of TTMP to the fermentation system in order to increase the TTMP yield [7,14]. Xiao et al. created a new two-stage temperature control method for TTMP production. Firstly, glucose was used to synthesize acetoin (the precursor of TTMP) with *Bacillus* at 37 °C, pH 7.0 ± 0.2, a stirring rate of 500 rpm, and airflow of 1.0 vvm in a fermentation tank. Then, 67.7 g/L of diammonium phosphate was added to the fermentation broth. Secondly, the fermentation broth was transferred to another reactor at 95 °C. After 2.5 h of spontaneous reaction, 8.34 g/L of TTMP was obtained [15]. This is an effective way to increase TTMP production while avoiding the toxic effect of a high concentration of diammonium phosphate on cells during the growth process of bacteria. Zhu et al. discussed the effect of pH on TTMP synthesis and found that weak acidic conditions were conducive to cell growth and the accumulation of precursors, while TTMP could be more effectively synthesized with an initial neutral pH in *B. subtilis*. The results showed that the optimal pH value for cell growth and precursor accumulation was 5.5, while the optimal pH value for TTMP production was 7.0. After 48 h of cultivation, the pH of the culture was transferred from 5.5 to 7.0, and the TTMP yield increased by 22.2% compared to that under the constant pH. The final TTMP yield reached 7.43 g/L [16].

Microbial fermentation has advantages such as natural and green products, low pollution, and a mild reaction. The production of TTMP by microbial fermentation has become a hot research topic in recent years. However, in order to achieve the industrialization and long-term development of TTMP production by microbial fermentation, the development of inexpensive fermentation substrates is conducive to reducing costs and increasing product profitability. Combined with a two-step fermentation strategy, Hao et al. used wheat bran as a fermentation substrate. Firstly, they synthesized the precursor, and then they added diammonium phosphate, which increased TTMP production by 6.8 times [17]. Liu et al. used soybean as a fermentation substrate to produce TTMP [18].

Nitrogen sources are essential nutrients for microbial growth that participate in many intracellular metabolic processes, regulate the ratio of intracellular primary metabolites to secondary metabolites, control the growth rate of microorganisms, and influence the production of products and the consumption of substrates. According to their nature, they can be divided into organic and inorganic nitrogen sources. Organic nitrogen sources account for a higher proportion and play a more critical role, as they are directly involved in the transamination and deamination of microorganisms in vivo. Organic nitrogen sources have a slower utilization rate compared to inorganic nitrogen sources.

The microbial production of TTMP is mainly based on glucose, which undergoes a series of biochemical reactions to produce acetoin in microorganisms. In the early stage of fermentation, large quantities of glucose are consumed to produce acetoin [19]. Therefore, molasses with a high sugar content, a by-product of the food industry, was chosen as the carbon source for fermentation in this study. Molasses is rich in fermentable sugars and is a carbon source with high quality and low cost [20].

Purification is necessary in order to use TTMP. The solubility of TTMP is very sensitive to temperature. When the temperature is 37 °C, the solubility is 4.77 g/L. However, when the temperature is 4 °C, the solubility of TTMP in water is 1.06 g/L. At low temperatures, the solubility is relatively low. Therefore, separating TTMP from complex fermentation broth is relatively simple. The fermentation broth is directly cooled to precipitate the TTMP, and it is then filtered and washed with ice water to obtain TTMP crystals with 99% purity [21]. Liquid fermentation is more conducive to sample separation. Xiao et al. proposed a method to recover TTMP: fermentation broth was evaporated at 60 °C under vacuum to yield a clean light yellow solution. In the interim, some white crystals emerged on the inner surface of the condenser. The solution was cooled to 4 °C before filtration using a precooled apparatus. All the crystals from the condenser wall and filtration were collected, recrystallized, and lyophilized. The product purity was 99.88%, which was determined by GC [9].

In this paper, by combining preliminary market research and an analysis of the metabolic pathway of microbial TTMP synthesis, molasses, a by-product of the sugar industry, was selected as the carbon source, and soybean meal (a by-product of oil extraction) was used as the nitrogen source. Molasses is viscous and dark in color with an aroma of yeast and sugar, and it contains 40–60% sugar [22]. Molasses has been recognized as an economic raw material for the production of high-value bioproducts, such as citric acid and succinic acid [23]. Soybean is a popular oilseed crop around the world, and its production is increasing at a faster rate because the demand for food and oil is increasing quickly at present. Soybean meal is a by-product of the production of soybean oil, which has a high protein content, and it is widely used to manufacture animal feed and produce soy concentrate and antibiotics [24,25]. Soybean meal is the main economic component of soybean and accounts for 60% of its value [26]. Soybean meal, a cheap source of organic nitrogen, is a fermentation substrate with great potential.

In our previous study, the ability of a cheap carbon source (molasses) to produce TTMP was investigated [27]. The highest TTMP yield (895.13 mg/L) was obtained after 36 h of fermentation with molasses as the carbon source and tryptone as the organic nitrogen source. In this study, the feasibility of using by-products of the food industry (molasses and soybean meal) to replace carbon and nitrogen sources (glucose and tryptone) for TTMP fermentation was further explored.

## 2. Results and Discussion

### 2.1. Composition of Soybean Meal

To effectively utilize soybean meal, it is vital to understand its chemical composition. The composition of the soybean meal used in this study is shown in Table 1. The soybean meal includes a crude protein content of 45.91%, total sugar of 14.82%, water of 10.45%, lipid of 4.62%, crude fiber of 9.65%, ash of 6.08%, and reducing sugar of 0.96%.

### 2.2. Pretreatment of Soybean Meal

Soybean meal is emerging as a cheap nitrogen source which is often used for solid-state fermentation by microorganisms. As a nitrogen source for fermentation, soybean meal has been studied for a long time. Zhu et al. [28] used defatted soybean meal and sodium nitrate as nitrogen sources to ferment arachidonic acid and obtained an optimum yield of 1.47 g/L. In a study on the production of 2,3-butanediol by *Bacillus licheniformis* BL1, Das et al. found that defatted soybean meal and soybean meal hydrolysate were able to increase the yield compared to conventional nitrogen sources [29]. Xiao et al. used molasses and soybean meal hydrolysate for the fermentation of acetoin and achieved a high acetoin content [30]. The fermentation potential of soybean meal for TTMP production was also tested in several studies. Besson et al. used soybean for the solid-state fermentation of TTMP and obtained 2.5 g/kg TTMP after the addition of 90 g/kg acetoin to the soybean and 14 d of fermentation [7]. Xiao et al. used soybean meal alone to replace a conventional nitrogen source for TTMP fermentation [9]. These previous studies illustrate that soybean meal is a popular and excellent nitrogen source for the fermentation of TTMP compared to conventional nitrogen sources.

**Figure 1:**
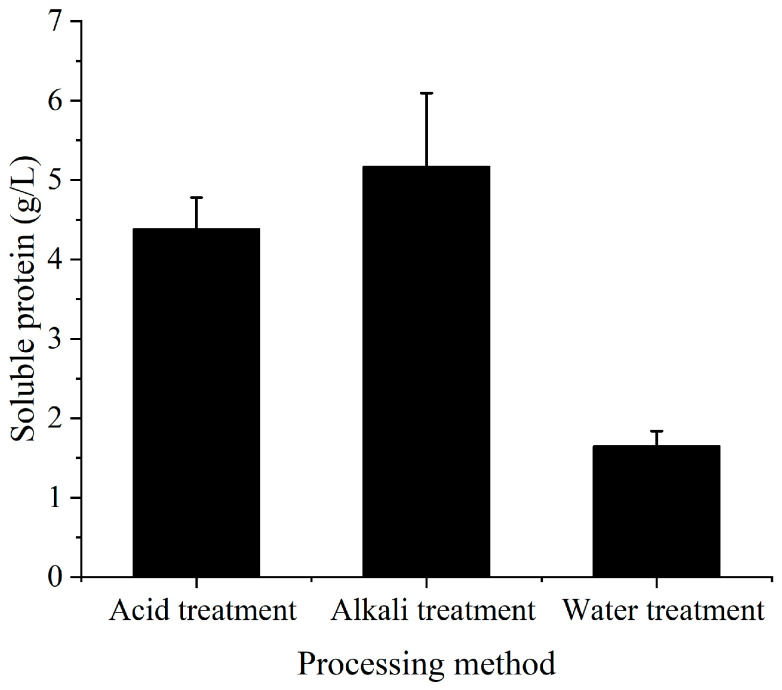
Soluble protein content of the supernatant from soybean meal treated using different methods.

**Figure 2:**
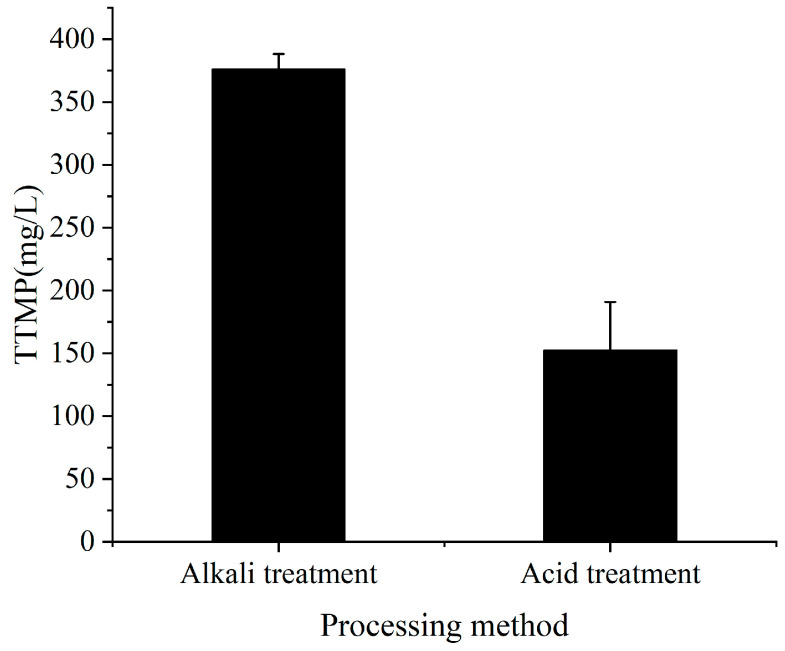
Production of TTMP by fermentation of the supernatant from soybean meal treated using different methods.

**Figure 3:**
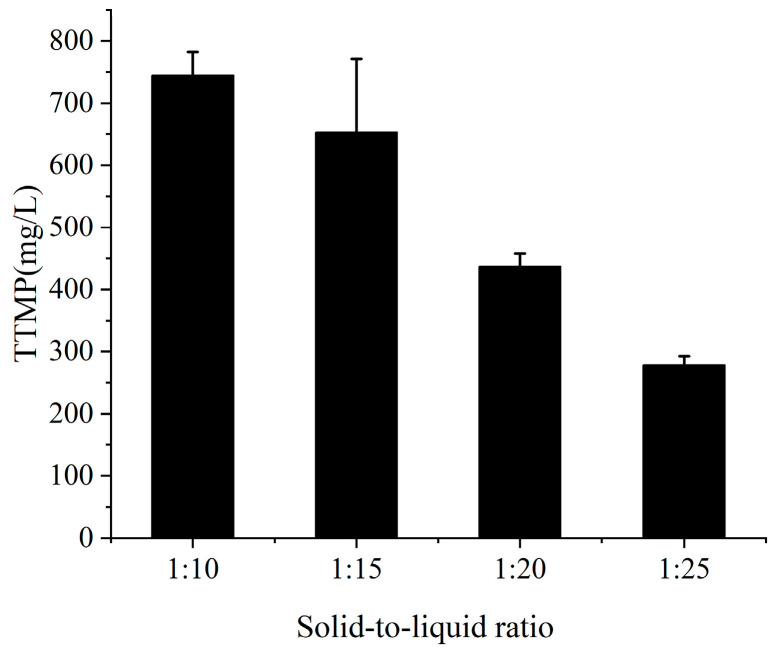
Effect of solid-to-liquid ratio on the TTMP yield using alkali-treated soybean meal.

In this study, three simple methods were selected to pretreat soybean meal so that the microorganisms could effectively utilize soybean meal for fermentation. Acid hydrolysate was the supernatant when the soybean meal was treated with 0.5 M HCl, and alkaline hydrolysate was obtained when the soybean meal was treated with NaOH. The soluble protein contents of three kinds of hydrolysates were determined by staining with Coomassie brilliant blue G250 (Figure 1). The results showed that the protein content from the water treatment was significantly lower than those of the other two treatments, but the difference in protein content between the acid and alkaline treatments was not significant. Therefore, the original nitrogen source (tryptone) was substituted by acid-treated and alkaline-treated hydrolysates, and the original carbon source (glucose) was substituted by treated molasses for co-fermentation; the contents of TTMP in the fermentation broth were measured (Figure 2). The results showed that alkali-treated soybean meal hydrolysate and treated molasses performed better together and produced more than twice as much TTMP as the acid-treated soybean meal hydrolysate. Therefore, in subsequent experiments, the alkali treatment was chosen as the pretreatment method for soybean meal. The effect of the solid-to-liquid ratio on fermentation was analyzed (Figure 3). The results showed that the highest TTMP yield was obtained with a solid-to-liquid ratio of 1:10 (soybean meal:water).

The final processes for the pretreatment of soybean meal were as follows: the soybean meal was ground and passed through a 100 mesh sieve. It was dissolved in distilled water at a solid-to-liquid ratio of 1:10 (soybean meal: water) to make a mixture, and the pH was adjusted to 8 with 2 M NaOH. The mixture was boiled for 30 min, and the pH was adjusted to 7. It was then centrifuged at 10,000× *g* for 10 min, and the supernatant was saved for use.

### 2.3. Determination of the Range of Each Component

**Figure 4:**
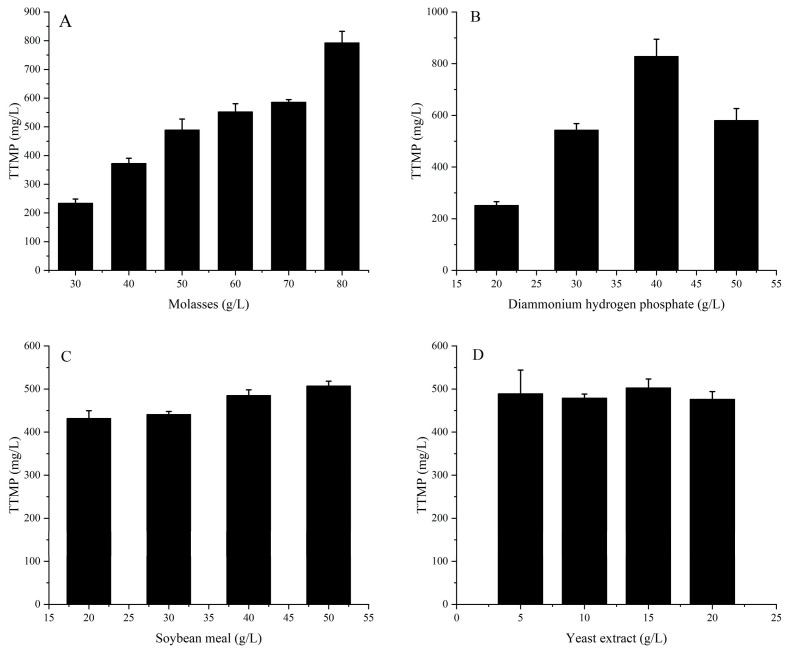
Effect of the content of each component on the yield of TTMP in fermentation medium. (**A**) Molasses content; (**B**) diammonium hydrogen phosphate content; (**C**) soybean meal content; (**D**) yeast extract content.

The molasses content ranged from 30 g/L to 80 g/L, and the fermentation results are shown in Figure 4A. The TTMP content increased when the molasses content increased. Therefore, 70–80 g/L was chosen as the range for response surface design. Ammonium is an important precursor of TTMP synthesis. The reaction of acetoin and ammonium (or ammonia) produces α-hydroxyimine, which is further converted to 2-amino-3-butanone and TTMP [8,18]. As an ammonium salt, DAP is widely utilized for TTMP fermentation [10,15]. Therefore, DAP was chosen as the inorganic nitrogen source in this study. The amount of DAP added to the medium was set at 20, 30, 40, and 50 g/L. The fermentation results are shown in Figure 4B. The TTMP content increased with the increasing DAP content when the DAP content was lower than 40 g/L and decreased when the DAP content was higher than 40 g/L. This phenomenon might be due to the toxic effect of a high concentration of DAP on the cells. In order to investigate the optimal DAP content, the range of DAP content was set at 30–50 g/L for the response surface design.

Zhu et al. confirmed that the use of soybean meal and yeast powder as a mixed nitrogen source was effective at increasing the accumulation of acetoin and TTMP [31]. Soybean meal hydrolysate and yeast powder were also chosen as the components of the medium in this experiment. The levels of soybean meal hydrolysate were chosen to be 20, 30, 40, and 50 g/L. The level of TTMP increased when the level of soybean meal increased (Figure 4C), so in subsequent experiments, the level of soybean meal hydrolysate was set at 40–50 g/L. As shown in Figure 4D, with the increase in the amount of yeast powder, there was no significant increase in the amount of TTMP. Therefore, in order to reduce the cost of fermentation, the minimum content of yeast powder (5 g/L) was selected in subsequent experiments.

### 2.4. Central Composite Design of the Response Surface Method

#### 2.4.1. Regression Model and Statistical Test

A total of 20 groups of experiments were conducted using CCD to optimize the TTMP fermentation medium, and the TTMP yields are shown in Table 1. The second-order polynomial regression equation showed the relationship between TTMP yield (Y) and three variables: molasses content (A), DAP content (B), and soybean meal content (C).

Y = 1312.36 + 43.76A − 141.47B + 43.06C + 53.96AB − 29.56AC + 6.53BC − 20.29A^2^ − 300.20B^2^ + 23.23C^2^

The predicted yield of TTMP (mg/L) was calculated using the above equation and is shown in Table 2. The residual between the predicted and experimental (actual) values was small. Table 2 shows that the experimental error was smaller, and the model was effective.

**Figure 5:**
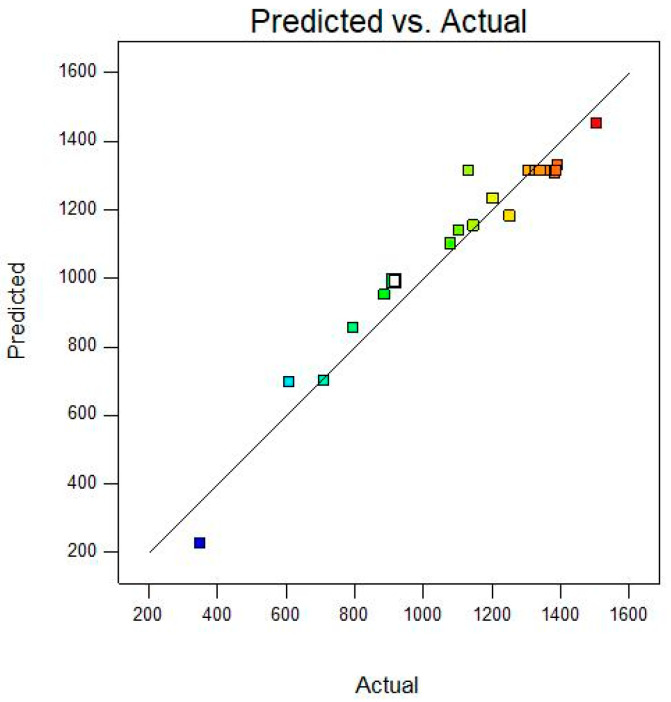
Comparison of the predicted and actual response values. The straight line indicates that the theoretical value was roughly equal to the experimental value.

The analysis of variance (ANOVA) for the model is shown in Table 3. It shows that the F value of the model was 19.18, *p* < 0.0001, indicating that the model was significant (*p* < 0.05). Moreover, for the lack of fit, the *p* value was 0.3635 (*p* > 0.05), indicating that the lack of fit was not significant and the model was fit for the demand of prediction. The coefficient of determination R^2^ (0.945) indicated the reliability of the model. The model could be used to analyze and predict the effect of the content of each component on the TTMP yield. Figure 5 shows the actual and predicted TTMP yields, which indicated a smaller gap between the experimental and predicted values.

#### 2.4.2. Graphics Analysis

**Figure 6:**
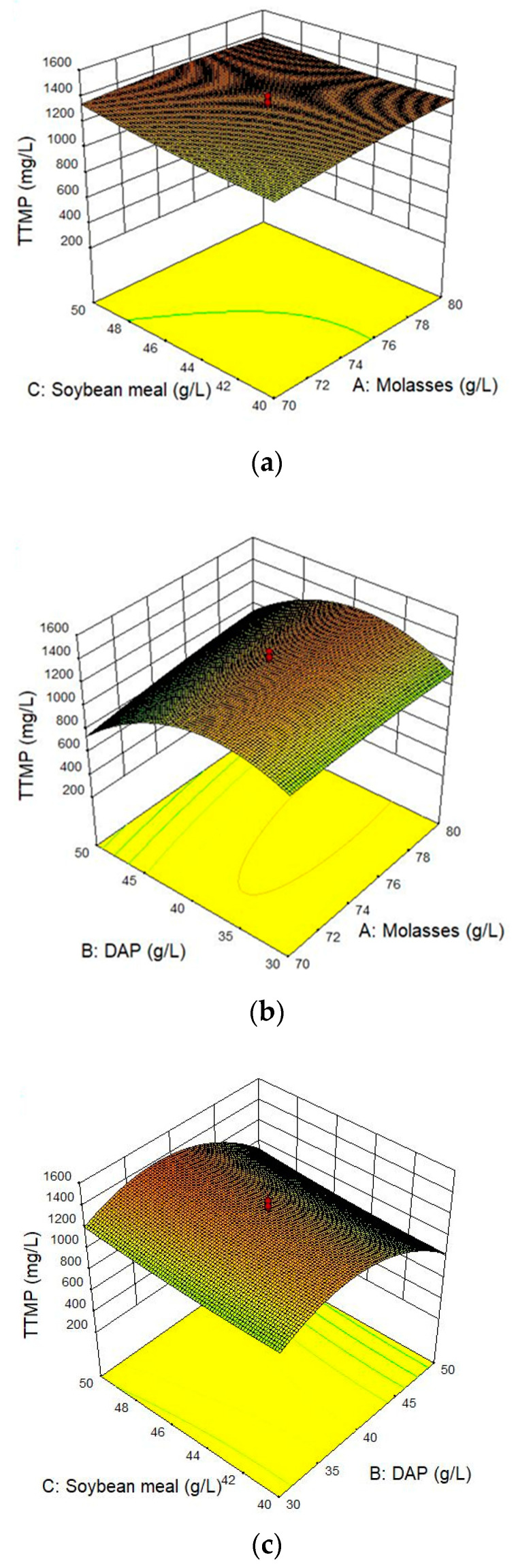
Response using CCD obtained by plotting: (**a**) molasses content and soybean meal content; (**b**) molasses content and DAP content; (**c**) soybean meal content and DAP content.

The interaction effects of these three compositions on the TTMP yield are shown through three-dimensional (3-D) response surface plots (Figure 6). The steeper slope indicates a more significant interaction between the two variables. In addition, a preliminary determination can be made from the color of response surface plot, which tends to deepen when the trend of change appears dramatically.

Figure 6a shows the effects of molasses content (A) and soybean meal content (C) on the TTMP yield. The effects of increased molasses content and soybean meal content on the TTMP yield were not significant. This phenomenon indicated that the interaction between molasses content (A) and soybean meal content (C) had a small effect on the TTMP yield. Figure 6b shows a convex 3-D graph demonstrating the interaction between DPA content (B) and molasses content (A) with a dense, elliptical contour, indicating that the model had a stable point of maximum value within this range. Figure 6c shows a 3-D graph demonstrating the interaction between DPA content (B) and soybean meal content (C). The TTMP production increased and then decreased slowly when the DPA content increased, while the TTMP production did not change significantly when the DPA content remained constant, and the soybean meal content increased. This indicated that the DPA content had a greater effect on TTMP production than the soybean meal content.

#### 2.4.3. Response Surface Optimization and Validation

The optimum values of the influencing factors were obtained by an analysis of Design-Expert 10.0.4.0 software to achieve the maximum TTMP productivity. The optimum conditions were as follows: a molasses content of 72.5 g/L, a DAP content of 37.4 g/L, and a soybean meal content of 53.4 g/L. Under the optimum conditions, the theoretical yield of TTMP was 1469.03 mg/L (Table 4). The optimum TTMP yield under optimal conditions was verified. The actual maximum TTMP yield was 1328.95 mg/L (Table 4). In general, the actual maximum was consistent with the theoretical maximum.

## 3. Materials and Methods

### 3.1. Microorganism and Culture Conditions

*Bacillus* sp. TTMP20 was isolated from high-temperature Daqu, which had a high TTMP yield in our previous study. The cryopreserved strain TTMP20 was incubated at 37 °C/200 rpm for 12 h in growth medium that consisted of tryptone at 10 g/L, beef extract at 5 g/L, and NaCl at 5 g/L. The activated strain TTMP20 (2%, *v:v*) was inoculated into the fermentation medium. The composition of the initial fermentation medium for the CCD optimization consisted of molasses at 50 g/L, soybean meal at 30 g/L, yeast powder at 10 g/L, and DAP at 30 g/L. In this study, batch fermentation was conducted in a 50 mL conical flask that contained 20 mL liquid medium (pH of 7.2, speed of 200 rpm, and a total fermentation time of 36 h). The fermentation conditions were 47 °C for 24 h and 60 °C for 12 h. The initial fermentation medium (without by-products from the food industry) was composed of glucose at 50 g/L, yeast powder at 10 g/L, tryptone at 30 g/L, and DAP at 30 g/L in our laboratory. The fermentation medium (with molasses instead of glucose) was composed of molasses at 150 g/L, yeast powder at 10 g/L, tryptone at 30 g/L, and DAP at 30 g/L in our previously published study in *Molecules*.

### 3.2. Pretreatment of Molasses

Cane molasses was diluted with purified water (1:1, m:m), boiled for 30 min, then cooled down rapidly with ice, and the pH was adjusted to 1.0–2.0 with concentrated sulfuric acid. The mixture was then left overnight. After centrifugation, the supernatant was adjusted to pH 6.0 using calcium hydroxide, kept at 60–70 °C for 30 min, and centrifuged (8000× *g* for 10 min). The supernatant was treated with 0.8% of activated carbon to adsorb the pigments for fermentation.

### 3.3. Pretreatment of Soybean Meal

(1) Acid treatment: Soybean meal was ground and passed through a 100 mesh sieve. A total of 5 g of soybean meal was treated with 100 mL of 0.5 M hydrochloric acid. The steps were as follows: The mixture was incubated at 60 °C for 3 h and then at 100 °C for 1 h, the pH was adjusted to 7, and it was centrifuged at 10,000× *g* for 10 min. The supernatant was saved for use.

(2) Alkali treatment: After treatment with a 100 mesh sieve, 5 g of soybean meal was mixed and 100 mL of water was added. The pH was adjusted to 8 with 2 M NaOH, the mixture was boiled for 30 min, and the pH was adjusted to 7. The mixture was centrifuged at 10,000× *g* for 10 min, and the supernatant was saved for use.

(3) Water treatment: After treatment with a 100 mesh sieve, 5 g of soybean meal was added to 50 mL of water, boiled for 30 min, and centrifuged at 10,000× *g* for 10 min. The supernatant was saved for use.

### 3.4. Analytical Methods

The TTMP content was determined using high-performance liquid chromatography (HPLC). An Agilent 5 TC-C18 (2) C18 Column (4.6 mm × 250 mm) and mobile phase (methanol:water = 70:30) were used for the separation. The flow rate was controlled at 0.5 mL/min; the column temperature was 37 °C, and the detection wavelength was 290 nm. The TTMP standard was dissolved in methanol and diluted to different concentrations to produce a standard curve. Cells from the fermentation broth were removed by centrifugation at 12,000× *g* for 15 min. The supernatant was diluted 10 times for an analysis by HPLC. The soluble protein content in the soybean meal hydrolysate was determined by staining with Coomassie brilliant blue G250.

### 3.5. Confirmation of the Range of Medium Component

The components of the fermentation medium included molasses, soybean meal, DAP, and yeast powder. To obtain a more accurate response surface model, the accurate range of each component in medium was determined first. The initial ranges of molasses content, soybean meal content, DAP content, and yeast powder content were 30–80 g/L, 20–50 g/L, 20–50 g/L, and 5–20 g/L, respectively. In a single-factor experiment, the concentrations of the other components remained unchanged.

### 3.6. CCD Design

Using the molasses content, soybean meal content, and DAP content as influence factors, and the TTMP yield produced by *Bacillus* sp. TTMP20 as the response value, the CCD design was realized using the software Design-Expert 10.0.4.0 (Table 5). After obtaining the actual response values, a multivariable equation was established, 3-D surface plots were drawn, and the interactions among the influencing factors were examined. Three replicates were performed for each group.

## 4. Conclusions

In our previous study published in *Molecules*, the production of TTMP using molasses instead of glucose achieved good results, but the maximum TTMP yield did not exceed 1000 mg/L [27]. In this study, the use of molasses instead of glucose as the carbon source and soybean meal instead of tryptone as the nitrogen source resulted in a maximum yield of TTMP that reached 1328.95 mg/L. This paper shows the great potential of pretreated by-products from the food industry as fermentation substrates. In this study, TTMP was produced with simply pretreated molasses and soybean meal as the fermentation substrates, and the content of each component in the fermentation medium was optimized by a single-factor experiment and the response surface method to obtain the optimal medium composition: molasses of 72.5 g/L, DAP of 37.4 g/L, soybean meal of 53.4 g/L, and yeast powder of 5 g/L. The optimal TTMP content was 1328.95 mg/L. This study can be summarized as follows: Considering the protein content and TTMP yield, alkaline treatment with a solid-to-liquid ratio of 1:10 (soybean meal:water) was the most suitable for soybean meal. The range of each component (molasses content, DAP content, soybean meal content, and yeast powder content) was then determined in order to design the CCD. At last, the values of three influencing factors (molasses content, DAP content, and soybean meal content) were optimized using the CCD design. Under optimum conditions (molasses of 72.5 g/L, DAP of 37.4 g/L, soybean meal of 53.4 g/L, and yeast powder of 5 g/L), the actual maximum TTMP yield (1328.95 mg/L) in this study was much higher than the TTMP yield (895.13 mg/L) under the conditions (molasses of 150 g/L, DAP of 30 g/L, tryptone of 30 g/L, and yeast powder of 10 g/L) in our previous study published in *Molecules*. In this study, with decreased molasses (more than half), decreased yeast powder (half), and a by-product of soybean meal instead of tryptone, the TTMP yield improved by 48.46% compared with our previous study. In summary, the cheaper fermentation medium had a much higher TTMP yield in this study, which improved the application potential of *Bacillus* sp. TTMP20.

## Figures and Tables

**Table 1:** Chemical composition of soybean meal.

Composition	Content
Total sugars	14.82%
Reducing sugars	0.96%
Ash	6.08%
Water	10.45%
Crude proteins	45.91%
Lipids	4.62%
Crude fiber	9.65%

Note: Table 1 was based on the fresh weight of soybean meal.

**Table 2:** Experimental results of CCD design.

Group	A (Molasses Content, g/L)	B (DAP Content, g/L)	C (Soybean Meal Content, g/L)	Actual Value (Y, mg/L)	Predicted Value (Y, mg/L)
1	80	30	40	1103.81	1139.4
2	70	30	40	1080.51	1100.68
3	70	50	40	609.254	696.77
4	80	30	50	1146.54	1153.35
5	75	40	45	1388.02	1312.36
6	75	40	45	1132.09	1312.36
7	75	23.1821	45	710.367	701.19
8	66.591	40	45	1252.31	1181.38
9	75	40	45	1356.15	1312.36
10	75	40	45	1307.46	1312.36
11	75	40	45	1326.65	1312.36
12	75	40	36.591	1383.98	1305.63
13	80	50	50	917.234	991.38
14	80	50	40	886.345	951.31
15	75	40	45	1340.89	1312.36
16	70	50	50	796.34	855.07
17	75	56.8179	45	349.557	225.35
18	75	40	53.409	1505.51	1450.48
19	83.409	40	45	1391.03	1328.57
20	70	30	50	1203.51	1232.86

**Table 3:** Analysis of variance for the model.

Source	Sum of Squares	d. f.	Mean Square	F-Value	*p*-Value	Significance
Model	1,695,000.00	9	188,400.00	19.18	<0.0001	**
A	26,151.55	1	26,151.55	2.66	0.1338	
B	273,300.00	1	273,300.00	27.83	0.0004	**
C	25,325.40	1	25,325.40	2.58	0.1394	
AB	23,291.19	1	23,291.19	2.37	0.1546	
AC	6989.07	1	6989.07	0.71	0.4186	
BC	341.11	1	341.11	0.035	0.8559	
A^2^	5931.48	1	5931.48	0.6	0.455	
B^2^	1,299,000.00	1	1,299,000.00	132.25	<0.0001	**
C^2^	7774.66	1	7774.66	0.79	0.3945	
Residual	98,198.97	10	9819.90	-	-	
Lack of fit	57,103.39	5	11,420.68	1.39	0.3635	
Pure error	41,095.58	5	8219.12	-	-	
Sum	1,794,000.00	19	-	-	-	
R^2^	0.945	-	-	-	-	
Adj R^2^	0.90	-	-	-	-	
Precision	17.48	-	-	-	-	

Note: The symbol ** (*p* < 0.01) represents an extremely significant difference. - represents no data for this item, and d. f. represents degrees of freedom.

**Table 4:** Optimization of fermentation medium for TTMP production.

Molasses Content (g/L)	DAP Content (g/L)	Soybean Meal Content (g/L)	TTMP (mg/L)	
			Predicted Value	Experimental Value
72.5	37.4	53.4	1469.03	1328.95

**Table 5:** The levels of three variables in this study.

Factors	Levels				
	−α	−1	0	+1	+α
(A) Molasses content (g/L)	66.59	70	75	80	83.41
(B) DAP content (g/L)	23.18	30	40	50	56.82
(C) Soybean meal content (g/L)	36.59	40	45	50	53.41

## Data Availability

All data generated or analyzed during this study are included in this published article.

## Sample Availability

Samples of the compounds tetramethylpyrazine are available from the authors.

## Abbreviations

TTMP	Tetramethylpyrazine
DAP	Diammonium hydrogen phosphate
